# Improved recording of work relatedness during patient consultations in occupational primary health care: a cluster randomized controlled trial using routine data

**DOI:** 10.1186/s13063-020-4168-8

**Published:** 2020-03-12

**Authors:** Salla Atkins, Tiia Reho, Nina Talola, Markku Sumanen, Mervi Viljamaa, Jukka Uitti

**Affiliations:** 1 New Social Research and Faculty of Social Sciences, Tampere University, Tampere, Finland; 2 Department of Global Public Health, Karolinska Institutet, Stockholm, Sweden; 3 Faculty of Medicine and Health Technology, Tampere University, Tampere, Finland; 4 Pihlajalinna Työterveys, Tampere, Finland; 5 Church of Finland, Tampere, Finland; 6 Finnish Institute of Occupational Health, Tampere, Finland; 7 Occupational medicine clinic, Tampere University Hospital, Tampere, Finland

**Keywords:** Occupational health, Randomized controlled trials, Sickness absence, Work-related illnesses

## Abstract

**Background:**

Prolonging working careers is a key policy goal in ageing populations in Europe, but reaching this goal is complex. Occupational health services are in the best position to contribute towards prolonging working careers through preventing illnesses that cause work disability and early retirement. However, impacting on the trajectory between illness and work disability requires continuity of care and follow up, enabled through identifying patients at risk. We aimed to determine whether a combined educational and electronic reminder system in occupational health care could improve the recording and follow up of primary care visits made by patients at risk of work disability, and whether the system could impact on sickness absence rates.

**Methods:**

This study is a pragmatic, cluster-randomized controlled trial using medical record data. Twenty-two Pihlajalinna Työterveys units were randomized into an intervention group receiving education and electronic reminders or a group receiving usual care through minimization methods. Patient consultation data were extracted from routine Pihlajalinna Työterveys patient registers from 2015 to 2017. In addition, process indicators were collected from the electronic system. Data were cleaned and analysed on an intention-to-treat basis using analysis of covariance.

**Results:**

There was no significant difference between intervention and control units in terms of sickness absences of different duration. Process indicators suggested that there was a change in physicians’ practice of recording patients’ risk of work disability and work-relatedness of visits following the educational intervention.

**Conclusion:**

Education with an electronic reminder can change physicians’ practice, but long-term follow up is needed to determine whether this impacts on patients’ sickness absences.

**Trial registration:**

ISRCTN Registry: ISRCTN45728263. Registered on 12 April 2016.

## Background

Prolonging working careers is a key policy goal among ageing populations in Europe [1, 2], but reaching this goal is complex. Economics and personal health influence decisions about whether to continue at work at pension age [3]. A more pressing problem in European settings is the increasing number of disability pensions, which at least in Finland mostly affects young people of working age [4]. An estimated 145000 [5] individuals are on early disability pension in Finland. Being on disability pensions affects personal finances [4], wellbeing [6] and national insurance expenditure. In 2015 the disability pension expenditure in Finland was 2057 million euros [7].

Poor working conditions and workplace risks increase the likelihood of disability pensions [8]. While work can bring economic, psychological and even health benefits [9], workplace risks and conditions can impact negatively on both physical and mental health [10, 11] and exacerbate already existing conditions, such as depression. Work-related disorders, and thus work disability, can be prevented through close collaboration between workplaces and health care. In Finland, occupational health services (OHS) provide both preventive services in the workplace and curative primary healthcare services for individual employees [12]. The key role of Finnish OHS is to provide healthcare services to organisations’ employees, during which they can identify patients at risk of work-related diseases, and identify injuries and illnesses that threaten an employee’s ability to work [13]. They are in a key position in the country to implement workplace interventions that can help prevent work-related diseases and decrease rates of work disability [14]. However, to date, little research has been conducted on the effectiveness of OHS in preventing work disability. In order to impact on work disability, we need to test different interventions for identifying patients at risk and to target prevention and care to them before their health worsens to the point of disability. The current OHS process for impacting on work disability mandates that a patient from primary care is directed to appropriate workplace or personal interventions following the identification of a risk to the employee’s ability to work or a work-related illness. According to a Finnish survey, 25% of mens’ and 32% of womens’ occupational health primary care visits were work-related [15].

Therefore, accurately recording the work-relatedness of each visit and the patient’s potential risk of disability is important. While recording work-relatedness is standard practice across OHS in Finland, to date no studies have been published on assessing this process, nor has there been any study of how well physician records match true risks, and how well OHS follow up patients and conduct interventions to prevent work disability after the employee’s visit.

We hypothesized that clearer recording of work-relatedness at primary care visits and systematic recording of the work disability risk of individual patients, with systematic follow up and initiation of intervention to mitigate risks, could help to reduce work disability. The aim of this study was to evaluate an intervention designed to improve recording and follow up of OHS primary care visits and its impact on sickness absences.

## Methods

The intervention protocol was reported in full elsewhere (see [16]).

### Setting

The study was conducted in Pihlajalinna Työterveys, a large private OHS provider, which at the time of starting the study had 28 private healthcare units across Finland. Pihlajalinna Työterveys had approximately 68,370 employees on their register in 2015. The organisation went through several rounds of mergers and corporate acquisitions during the study period, which led to a substantial increase in patient and healthcare unit numbers.

### The intervention

The intervention was multifaceted and implemented sequentially. First, a notice was sent to the entire organisation informing all practitioners that the study would be conducted. The intervention consisted of two separate activities: one involved training, mentoring and follow up of physicians in intervention units on how to identify and record work-related illnesses during primary care visits and how to identify and record risk of work disability. The training sessions were conducted at each intervention unit. During the sessions the intervention and its components were introduced, and information about work-related illnesses was reinforced. This also included training on the intervention processes - actions that were to be initiated after a patient was identified during a visit as at risk of work disability. Following the training, a project physician responded to questions and followed up with training participants by telephone. Second, an organisation-wide change was made to the electronic healthcare system, clarifying the way in which work-relatedness and risk of disability pension was recorded. This change reinforced the messages given in the training. The electronic change was made to clarify language in sections where work disability risk was assessed. No specific training was conducted on the change to the electronic record system, as changes were minor and had been introduced in the intervention training. Therefore, intervention physicians were more likely to adhere to the change in the electronic health record, as they had been trained.

The trainees in the intervention sites were those occupational health physicians who were responsible for collaboration with their own patient companies, working at any of the 22 sites included in the study. The intervention and control sites had 58 physicians and 50 physicians, respectively, employed during the study. These physicians would be responsible for contacting workplaces, and would be involved in tailoring patients’ work tasks or conducting other workplace interventions.

If the occupation health (OH) physician noted a patient’s visit as being related to work or that the patient presented with a condition that could potentially result in work disability in the near future, they marked this onto an electronic system. Following this, a sequence of events was kicked off at the intervention sites. The OHS nurses responsible for the employer organisations used the electronic health records to collect the patients identified as at risk. Together with the physicians they then initiated the interventions that the physician recommended, either for the patients or more widely at the workplace. These interventions could include, for example, an occupational health collaborative negotiation to modify the employee’s work tasks or timing, or organisational interventions focused on workplace ergonomics or teamwork counselling. Other interventions could include, for example, starting medical or vocational rehabilitation for the individual patient, involving both the workplace and the patient/employee. It was not possible to collect the number of these interventions in this study, since the individual patients that were identified as at risk of work disability or that had a work-related condition could not be associated with the interventions conducted at workplace level. A fuller description of the intervention can be found in the Template for intervention description and replication (TIDIER) reporting guide for population health interventions: tidierguide.org/#/gen/pFqrFqw3M

Information about the study was sent to all sites in April 2016. The intervention training was conducted in May 2016. The electronic change to systems was implemented on 9 March 2017. Data collection ended in December 2017.

### Randomization

We included 22 Pihlajalinna Työterveys clinics that were functional in 2016 in the study. We treated each healthcare unit as a cluster, as individual randomization in this context would have been challenging. NT, the team statistician, conducted initial simple randomization to randomize the first four clusters. After this, we used the minimization approach to randomize the remaining 18 clusters so that the following confounders were balanced across the intervention and control sites (see [16]): (1) the occupational sector (e.g. industrial, service sector, public service), (2) presence of a large industry patient and (3) patient volume per site. The occupational health professionals and the research team were not blinded to the intervention.

### Outcomes

Our primary outcome was reduction of the mean number of medium-term (4–14 calendar days) of sickness absences per intervention and control centre from baseline after 1 year of follow up as measured from information in OHS patient records [16]. We considered medium-length sickness absences from 4 to 14 calendar days, instead of 9 working days as indicated in the original protocol. We chose this to match our findings more closely with the Finnish Insurance Agency’s definitions for sickness absences, which considers medium-length sickness absences as absences including 9 working days (including Saturdays but not Sundays). The patient records included also weekends as sickness absence days, which differed from this approach. A similar choice was made to that in a previous report [17].

Our secondary outcomes were:

1. Reduction in the mean number of short-term (1–3 consecutive calendar days) sickness absences from the workplace per cluster after 1 year from the start of the intervention as measured either by self-reported sickness absences recorded on the OHS system or sickness absences certified by OHS physicians working at the OHS units included
2. Reduction in the mean number of any form of work disability pensions as measured by an employee registered on the central pensions register as receiving a work disability pension from baseline to up to 2 years from the intervention
3. Reduction in the mean number of long-term (15 or more consecutive calendar days) sickness absences from the workplace per cluster from baseline to 1 year after the intervention as measured by OHS records

The follow-up time was set at 1 year due to funding and the planned study duration. This article focuses on reporting the primary outcome, medium-term sickness absences, as we deemed the follow-up period too short to report on disability pensions or long-term sickness absences. We also report short-term sickness absences and the process indicators collected on recording work relatedness and risk of work disability at consultations across the control and intervention sites.

### Power calculation

Our initial power calculation suggested that we would have 91% power to detect a 10% change in mean sickness absence rates across the intervention and control clusters, if we had 22 occupational health units with 24,892 patients. For the trial, we retained all 22 units, with 26,804 patients recorded on the system.

### Data collection

We collected medical record data on patients’ healthcare consultations at Pihlajalinna Työterveys from 2015 to 2017. The medical records included 68,370 patients in 2015 and 107,413 patients in 2017. The cohort was dynamic in that patients could be added to the cohort as the study progressed. Data were pseudonymised, and researchers had no access to patient-identifying data. All patients above the age of 18 years and whose employers had a contract with Pihlajalinna Työterveys including primary healthcare services were included in the study. The data were combined with pseudonymised data from the Finnish Centre for Pensions, where we obtained data on all participants’ pensions granted for the study period.

### Data analysis

After data collection was complete, we noted that Pihlajalinna’s acquisition of another large occupational health services provider impacted on our outcomes. Therefore, we used all initially randomized sites in the intention-to-treat analyses and excluded them in the per-protocol analyses.

We included data on curative patient visits to OHS physicians responsible for patient organisations. OHS services have many casual workers, who deal with primary care patients but are not occupational health specialists and most of them were not exposed to training. We also excluded preventive visits such as health examinations. We analysed data 6 months before the intervention, during the intervention, and for 6 months after the intervention. After initial analysis we chose a period of 6 months after the intervention corresponding with the same season of the 6 months preceding the intervention, to ensure that seasonal effects did not confound our analysis. We analysed data using analysis of covariance (ANCOVA), setting alpha at 0.05.

We also analysed process indicators among intervention and control clinics. These indicators included whether physicians had changed their practice of recording relatedness to work, during the consultation after the intervention. The intervention required a physician to record whether or not the patient’s visit was related to work or whether this was not assessed. We analysed changes after the educational intervention and after the change in the electronic system, using descriptive statistics.

## Results

**Fig. 1:**
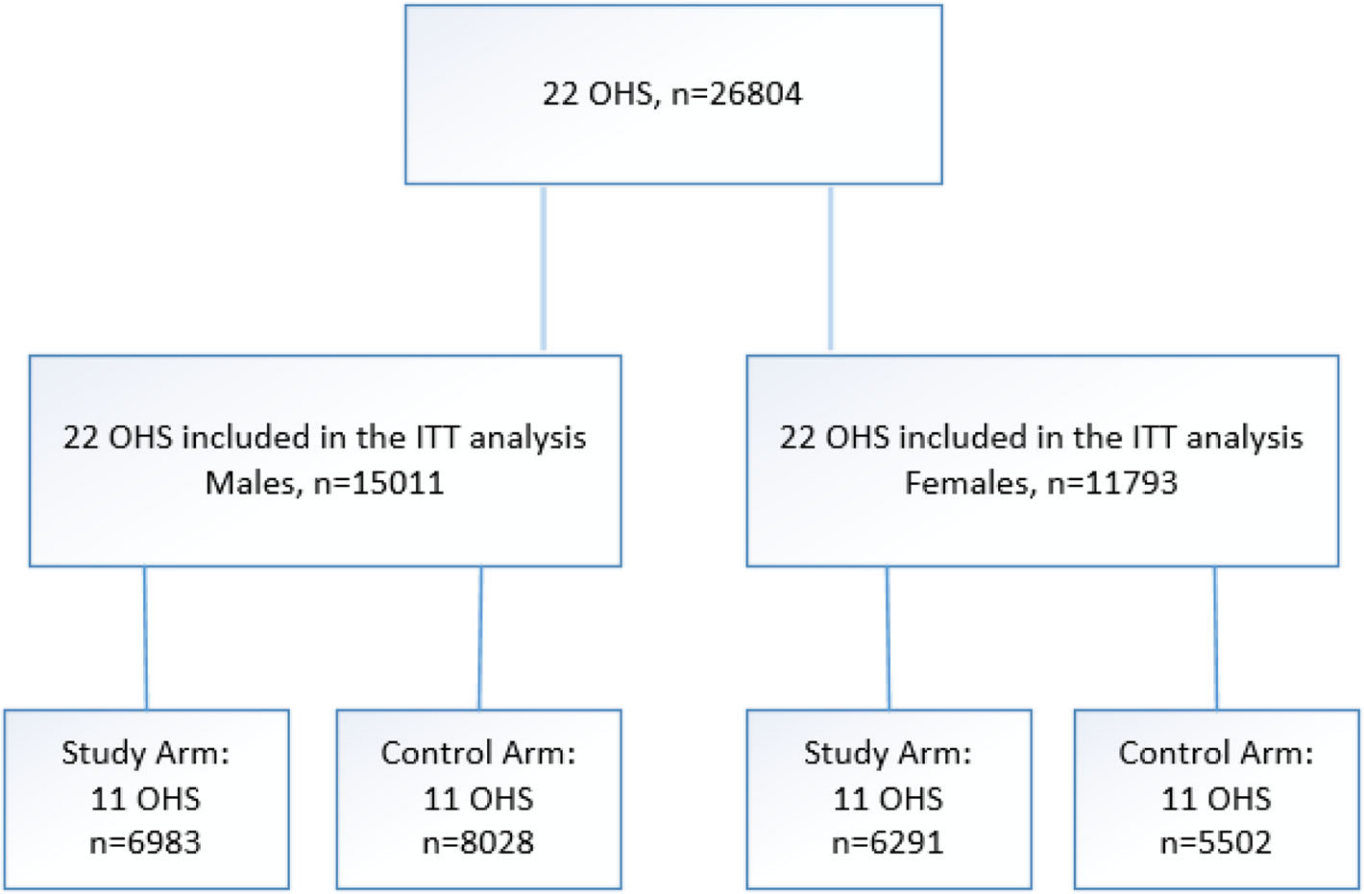
Flowchart of the trial randomization

The flowchart (Fig. 1) presents the final data after randomization, divided by sex.

Figure 1 Flowchart for the intention-to-treat analysis (ITT). OHS, occupational health services

The baseline characteristics of the study population are shown in Table 1.

**Table 1:** Baseline characteristics of intervention and control groups by sex: mean (standard deviation) or percentage (%) within group

	Women, *n* = 8735				Men, *n* = 11,192			
Baseline characteristics	Control group (*n* = 3911)		Intervention group (*n* = 4824)		Control group (*n* = 5828)		Intervention group (*n* = 5364)	
Age, mean (sd)	44	(12)	42	(12)	43	(12)	42	(12)
No sick leave, only visit, *n* (%)	1827	(47)	2038	(42)	2924	(50)	2480	(46)
Number of visits per person during 6 months preceding the intervention, mean (sd)	3	(2)	3	(2)	2	(2)	3	(2)
Any work disability pension^a^, *n* (%)	133	(3)	195	(4)	128	(2)	180	(3)
Primary outcome^b^								
Medium-term SA (4–14 days), *n* (%)	783	(20)	1116	(23)	1246	(21)	1127	(21)
Secondary outcome^b^								
Short-term SA (1–3 days), *n* (%)	1555	(40)	2087	(43)	1993	(34)	2056	(38)
Long-term SA (15+ days), *n* (%)	406	(10)	563	(12)	569	(10)	557	(10)
Number of SA episodes, mean (sd)	2.1	(1.6)	2.3	(1.8)	2.1	(1.6)	2.1	(1.6)
Total length of SA, days, mean (sd)	8	(21)	10	(28)	7	(23)	8	(23)

*SA* sickness absence

^a^Partial fixed-term disability pension, fixed-term disability pension, partial disability pension, permanent disability pension, vocational rehabilitation allowance

^b^Including only those with sick leave (control group, *n* = 4990; intervention group, *n* = 5668)

There were differences between women at the intervention and control sites in terms of age, proportion of registered employees visiting without sick leave, total number of visits, and medium-term and short-term sickness absences. In men, only age, visit without sick leave and short-term sick leave differed across the intervention and control sites (Table 1).

The results of our primary outcome analysis are shown in Table 2. As can be seen, the intervention had no significant effect on short-term, long-term or medium-term sickness absences in men or women.

**Table 2:** Intention-to-treat analysis, sickness absences before and after the intervention (*n* = 22)

Outcome variable Men	Baseline 6 months before the intervention 1.5.2015–31.10.2015		6 months after the intervention 1.5.2017–31.10.2017		Main comparison between intervention and control groups (CI 95%)	
	Control unit	Intervention unit	Control unit	Intervention unit		
	*n* = 11	*n* = 11	*n* = 11	*n* = 11		
Primary outcome	mean (sd)	mean (sd)	mean (sd)	mean (sd)		
Medium-term SA (4–14 days)	187 (160)	151 (111)	201 (176)	149 (70)	22	− 46 to 91
Secondary outcome						
Short-term SA (1–3 days)	316 (230)	319 (327)	381 (319)	317 (195)	66	− 122 to 255
Long-term SA (15+ days)	94 (63)	84 (48)	99 (65)	81 (45)	10	− 23 to 43
Total length of SA, days	4378 (2751)	3886 (2396)	4755 (3486)	3834 (2075)	486	− 1118 to 2091
Number of SA episodes	598 (442)	554 (478)	681 (552)	547 (292)	104	− 177 to 384
Women						
Primary outcome	mean (sd)	mean (sd)	mean (sd)	mean (sd)		
Medium-term SA (4–14 days)	116 (96)	153 (156)	160 (122)	193 (138)	−16	− 123 to 91
Secondary outcome						
Short-term SA (1–3 days)	261 (242)	331 (399)	323 (243)	357 (178)	−18	− 201 to 165
Long-term SA (15+ days)	68 (74)	93 (85)	89 (75)	105 (62)	−3	− 52 to 47
Total length of SA, days	3041 (3116)	4172 (4161)	3978 (3405)	4772 (2952)	− 233	− 2655 to 2189
Number of SA episodes	445 (408)	577 (626)	571 (429)	655 (365)	− 41	− 372 to 290

*SA* sickness absence

Short-term and long-term sickness absences decreased among men and increased among women, though none of these changes were statistically significant. The per-protocol analysis, excluding entire occupational health units, had similar results.

Our analysis of process indicators that measured how intervention and control groups recorded patient visits in practice was more promising. Table 3 shows change in physicians’ practice, at baseline, after education, after the electronic information system change and 6 months after the intervention.

**Table 3:** Process indicators: physician registration of work-relatedness of each patient visit

Outcome variable	Baseline 6 months before 1.5.2015–31.10.2015				Intervention (education) 1.5.2016–8.3.2017				Change in the information system (electronic reminder) 9.3.2017–30.4.2017				6 months 1.5.2017–31.10.2017			
	Control unit		Intervention unit		Control unit		Intervention unit		Control unit		Intervention unit		Control unit		Intervention unit	
	*n* = 11		*n* = 11		*n* = 11		*n* = 11		*n* = 11		*n* = 11		*n* = 11		*n* = 11	
	*n*	%	*n*	%	*n*	%	*n*	%	*n*	%	*n*	%	*n*	%	*n*	%
Not assessed	2	0	365	3	10,119	50	1763	9	1918	61	308	10	5881	59	1463	13
Not related to work	10,888	89	11,389	85	7581	38	14,525	75	831	26	2348	74	3001	30	7785	72
Work-related	1311	11	1714	13	2375	12	3198	16	398	13	490	16	1124	11	1657	15
*Total*	*12,201*	*100*	*13,468*	*100*	*20,075*	*100*	*19,486*	*100*	*3147*	*100*	*3146*	*100*	*10,006*	*100*	*10,905*	*100*

Table 3 shows that before the intervention most visits were recorded as “not related to work”, which was the default setting (89% and 85% across control and intervention units, respectively). After the institutional information presented and education conducted at intervention units, a change was observed where 75% of intervention units’ records and 38% of control units’ records stated “not related to work”. At the same time, the rates of “not assessed” increased in both units, more in the control units (50%) than in the intervention units (9%).

However, after the electronic reminder in the system changed and the default setting changed to “not assessed” from “not related to work”, we can see that while the control sites’ default answers increased (from 50% to 61%), the intervention sites’ default answers stayed nearly the same (from 9 to 10%), suggesting that intervention sites’ recordings were actual recordings made actively by physicians more than default choices. These effects were sustained over time. As the physicians’ recording improved, we can see that the percentage of visits related to work also increased, from 13% in the beginning to 15% at the end. Trends in recording possible work disability were similar across intervention and control sites. Physicians recorded similar numbers of possible future work disability for each consultation across intervention and control sites. There were slightly more records of no threat of disability at the intervention sites than there were at control sites.

## Discussion

Though our intervention showed no effect on sickness absences, it produced a promising indication of the effectiveness of education in improving occupational health professionals’ practices of recording work-related visits in primary care. This effect was supported by a change in electronic information systems.

While there was no statistical difference between the intervention and control arms in the rates of sickness absence as primary and secondary outcomes, there may be a number of reasons for the lack of differences. First, while approximately 15% of visits were identified as work-related, these form a relatively small subset of the entire population analysed for detecting a difference in sickness absences. At the individual level, actions leading to shortening of sickness absences take time, as rehabilitative processes are gradual. In addition, initiating individual work modifications in collaboration with workplaces requires time. Second, many of the conditions that are work-related require workplace interventions starting with including workplace assessment, with subsequent commitment by employers to implement these changes. An example of such intervention could be improving workplace psychological wellbeing [18], or changing the workplace environment, for example lighting [19] or disruptions from open plan offices [20]. These interventions are considerable commitments for organisations both in terms of processes and financially, and may take time to be implemented. In order for our intervention to impact on these, it should have had a workplace outreach component.

The increase in sickness absences among women can be related to increasing age over time [21] but also to poor interpersonal relations at the workplace [22]. Our linked study on frequent attenders in occupational health services similarly identified women as at risk of frequent use of services [23], where particularly women from the service industry and public administration were at risk [23]. We also found that frequent attenders of OHS primary care are also at increased risk of sickness absences after their consultation frequency has diminished [24]. This supports the lack of impact on sickness absences, when no workplace intervention was included.

Finally, a possible reason for lack of impact is changes in national rules for sickness certification, where employees could be absent from work without a certificate for a longer period (from 3 days to 7 days), which was implemented by many businesses and public organisations [25] during the study period. These, and other changes in sickness certification over time are more likely to impact on sickness absence rates than our intervention. The study sites also experienced relatively high physician turnaround. This means that our intervention might not have reached all practising physicians in the intervention sites. This suggests a need for a continuous education approach in future interventions.

Our study has several strengths and limitations. As the Pihlajalinna patient register has a large, nationwide sample representing different industries, we can consider our sample generalizable to the working age population in Finland. However, our pragmatic trial approach meant that we could not control the fidelity with which physicians adhered to the educational programme, nor were we able to determine which activities increased at the workplaces after the intervention. We also could not prevent crossover of physicians from the intervention to the control arm. Nevertheless, conducting such trials using routine patient registers allowed us to evaluate the outcome of the intervention with a large sample of high-quality data.

Despite the intervention not impacting on patients’ sickness absences, the impact of the educational intervention is promising. Early identification of patients with work disability risk enables timely follow up by the OH team and early intervention for issues that might threaten ability to work. This can possibly improve continuity of care in primary healthcare settings. OHS physicians are seen as better positioned to evaluate the need for sickness absences than general practitioners working in other settings [26, 27], and early consultation with an OH physician has been found effective in reducing the total number of sickness absence days taken by individuals who are at risk of sickness absences [28]. With a simple educational intervention combined with an electronic reminder, data indicate that occupational health physicians in 11 intervention clinics changed their practice of recording work-relatedness and potential for work disability. These effects were sustained after the intervention was concluded. As recording in electronic systems is a challenge and poorly functioning electronic referral systems can even result in occupational stress [29], this is a positive and fairly surprising outcome. Clinicians may feel that electronic health records impact negatively on their professional satisfaction [30], therefore the systems need to be both meaningful and easy to use. This simple intervention succeeded in improving the accuracy and frequency of recording. This better and more accurate recording can enable better follow up, interventions and assessment. Further training can reinforce this message. While recording of work-relatedness at consultations or the patient’s risk of work disability itself does not translate into reduced sickness absences, there is a possibility that improved recording can result in better reporting to employers, and better and timelier opportunities for preventive actions in the workplace and for patients.

## Conclusion

Our cluster, pragmatic, randomized controlled trial using patient registers as data did not find that an educational and electronic health information intervention had significant effects on sickness absences in the context of occupational health primary care in Finland. However, the intervention changed occupational health physicians’ practice of recording the work-relatedness of patient consultations, and potentially enabled better continuity of care and follow up for patients at risk of needing disability pensions. In future, such interventions should include detailed follow up of patients, with a workplace component to ensure adequate follow up of and intervention for patients at risk.

## Data Availability

The datasets generated and/or analysed during the current study are not publicly available due to personal identifiers and sensitive medical record data, but are available de-identified from the research team on reasonable request.

## Abbreviations

OH: Occupational health
OHS: Occupational health services
SA: Sickness absence
